# The Late Dr. Mackay—Proceedings of the Medical Society

**Published:** 1867-07

**Authors:** 


### The late Dr. Mackay—Proceedings of the Medical Society.

There was a full attendance of the members of the Erie County Medical Society at the rooms of the Buffalo Medical Association, on Monday evening, the 8th instant, to express the sentiments of the Society at the loss of one of their oldest and most esteemed members, the late Edward Mackay, M. D.

The meeting was called to order by the President, Dr. J. R. Lothrop, who paid the following feeling tribute to the memory of the deceased:

*Gentlemen of the Erie County Medical Society:*

We are met together this evening to make observance of the loss of one of our oldest members. Dr. EDWARD MACKAY, after a long and weary illness, died yesterday at the age of fifty-seven years. Long resident here, well and widely known and respected; till within a few years active and useful in our midst, his death is a bereavement which we all share. To him, as to many another schooled in suffering, death brought welcome relief. To us who see a professional brother taken away in the prime of his powers and usefulness, the event seems most untimely.

Our departed friend was denied entrance upon that period, when, after the labors and cares of middle life, a physician may look to have rest. While yet in active manhood, in the midst of labor and in the full tide of success, he was suddenly brought down, almost helpless, to that bed of pain from which he was not again to rise.

The early period of his illness was cheered by a hope of recovery, which slowly faded away; and then the conviction grew stronger each day, that his life was drawing to a close. Disciplined by pain and lingering sickness, his mind was prepared to welcome the great change; and he cheerfully laid down the burden of a life which had become, indeed, weary.

The occasion calls upon us to pay a proper tribute to his virtues, and to honor his memory. My acquaintance with the deceased has been somewhat brief. I knew him for a few years only, as a busy and hard worked practitioner. I have known him now during several other and sadder years, under trial, suffering and disappointment. As he bore himself with becoming manliness in prosperity, so he bore his heavy afflictions with a becoming courage and cheerfulness. You who have known him longer, can speak of his life and qualities more in detail. But short as my acquaintance with him has been, it has been long enough to make known to me his uniform kindness and gentle bearing, his great practical skill, the general breadth and soundness of his attainments, and his many excellent qualities as a man and a physician. But not to anticipate what many of you can, from better knowledge, in more fitting words, express, I leave to you the sad, but not unpleasant office, of expressing in a manner, altogether worthy of the occasion, our tribute to the memory of the deceased, and our sympathy for the bereaved.

Dr. Strong said:

Mr. President:—I believe it to be true of our deceased friend, that being a foreigner by birth, and obtaining his education and spending most of his life in the most favored nations of Europe, he naturally had advantages from the acquaintance of distinguished medical men abroad far superior to most, if not to any of us. He was on familiar terms with distinguished men of London, Dublin, Edinburgh, Paris, and some of the great German cities. Speaking several (I believe four or five) European languages with fluency, some of them so finely as to make him undistinguishable from "one native and to the manner born." It is very common in this world that sickness and death are attended by circumstances of great and even peculiar trial and affliction. But the circumstances attending the inception and progress of the disease which has now terminated the life of our friend Mackay, were unwontedly trying to faith and patience, and rare in their afflictive character.

Dr. Mackay when laid aside, had arrived at that full maturity of his powers, when accurate diagnosis was made with facility, and successful prescribing was well nigh intuitive; when in a word the treatment of disease was performed with satisfaction, not only to patients, but to his own enlightened conscience. He had attained to a position in which he was reaping the rewards of faithful labor, in honor and pecuniary emolument, when he received the injury from which was dated his sickness and suffering. And then to be struck down with a disease which left the lower half of him, as it were, with his vital organs intact, with mind and heart, his sympathies and sensibilities keenly alive, all aggravated and intensified by the thought that there was little or no hope of amendment, and that he must drag out weary years at once painful, helpless and hopeless, I repeat, thus to be stricken and thus to suffer, made a case peculiar and rare in its kind and degree of afflictiveness to him, and most moving in its appeal to the sympathies of all who attended upon and saw him. To say that he bore up under the affliction of Providence with a patient, heroic spirit, is but giving utterance to what all knew and admired who were about him.

I have expressed to him in years gone by the regret I felt to see him so unfrequently at our professional meetings. It is with far deeper regret that we are never more to meet him here or elsewhere, except it be "beyond the river," which we must all prepare to cross.

After the addresses of Dr. Lothrop and Dr. Strong, the committee to whom the task had been assigned of preparing a brief sketch of the life and services of the deceased, and resolutions expressive of the sentiments of the Medical Society, made the following report:

Gentlemen of the Erie County Medical Society:

We meet together this evening on account of the death of one of our members, Dr. Edward Mackay, who has maintained a very important and influential position in the medical profession and in this community for nearly thirty years.

Dr. Mackay was born in Macksgrove, County of Cork, Ireland, February 17th, 1810, and has been engaged in active practice for nearly thirty-five years. He graduated at the College of Physicians, in Dublin, and was a Fellow of the Royal College of Physicians and Surgeons of Edinburgh. He also had a medical degree from the German University of Giesen, and another from one of the colleges in Paris. From 1835 to 1838 he was in the service of the English Contingent as surgeon of the Ninth Regiment Royal Lancers, in Spain, during the war of the Don Carlos rebellion. He came to the United States in 1838, and took up his residence in Buffalo in the autumn of the same year.

Cholera was prevailing at the time very severely, and Dr. Mackay found his services called into requisition. Such was his devotion, energy, skill, and success, that he at once took rank as an able and reliable practitioner, and as such was constantly engaged until attacked with the illness whose sad issue has called us together. For thirteen years he was one of the physicians to the Buffalo Hospital of the Sisters of Charity, the onerous duties of which position he discharged with the utmost fidelity until compelled by declining health to resign his post. In 1861 symptoms of paraplegia manifested themselves, and despite the early recognition of the disease, and the best directed efforts of his medical advisers, both in Europe and America, to arrest it, the disorder steadily progressed.

During its course he suffered excruciating and almost constant pain, and underwent a necessary, dangerous, and most formidable surgical operation, although known to be only palliative, with great resolution, patience and fortitude. Death, which he ardently prayed for, in the hope and trust of a blessed immortality, came to his relief July 7th, 1867. In view of this dispensation it is

*Resolved*, That the members of this Society and the medical profession in general, have great reason to deplore the loss of one of their fraternity whose talents and attainments commanded their respect and endeared him most justly to the very large number of our citizens of whom he was the professional attendant.

*Resolved*, That we most respectfully condole and sympathize with his afflicted family.

*Resolved*, That we will attend his funeral in a body, and wear the usual badge of mourning.

*Resolved*, That a copy of this statement and resolutions be transmitted to the family of the deceased, and be sent for publication in the *Buffalo Medical and Surgical Journal*, and the city papers.

